# Responses to Hypoxia and Endoplasmic Reticulum Stress Discriminate the Development of Vitreous and Floury Endosperms of Conventional Maize (*Zea mays*) Inbred Lines

**DOI:** 10.3389/fpls.2017.00557

**Published:** 2017-04-13

**Authors:** Mathieu Gayral, Khalil Elmorjani, Michèle Dalgalarrondo, Sandrine M. Balzergue, Stéphanie Pateyron, Marie-Hélène Morel, Sylvie Brunet, Laurent Linossier, Caroline Delluc, Bénédicte Bakan, Didier Marion

**Affiliations:** ^1^Biopolymers, Interactions, Assemblies, Institut National de la Recherche AgronomiqueNantes, France; ^2^POPS (transcriptOmic Platform of iPS2) Platform, Centre National de la Recherche Scientifique, Institute of Plant Sciences Paris Saclay, Institut National de la Recherche Agronomique, Université Paris-Sud, Université Evry, Université Paris-SaclayOrsay, France; ^3^Institute of Plant Sciences Paris-Saclay, Paris Diderot, Sorbonne Paris-CitéOrsay, France; ^4^Agropolymer Engineering and Emerging Technologies, Institut National de la Recherche AgronomiqueMontpellier, France; ^5^Limagrain Céréales IngrédientsRiom, France; ^6^Limagrain EuropeChappes, France

**Keywords:** maize, endosperm, vitreousness, transcriptome, hypoxia, unfolded protein response (UPR)

## Abstract

Major nutritional and agronomical issues relating to maize (*Zea mays*) grains depend on the vitreousness/hardness of its endosperm. To identify the corresponding molecular and cellular mechanisms, most studies have been conducted on opaque/floury mutants, and recently on Quality Protein Maize, a reversion of an *opaque2* mutation by modifier genes. These mutant lines are far from conventional maize crops. Therefore, a dent and a flint inbred line were chosen for analysis of the transcriptome, amino acid, and sugar metabolites of developing central and peripheral endosperm that is, the forthcoming floury and vitreous regions of mature seeds, respectively. The results suggested that the formation of endosperm vitreousness is clearly associated with significant differences in the responses of the endosperm to hypoxia and endoplasmic reticulum stress. This occurs through a coordinated regulation of energy metabolism and storage protein (i.e., zein) biosynthesis during the grain-filling period. Indeed, genes involved in the glycolysis and tricarboxylic acid cycle are up-regulated in the periphery, while genes involved in alanine, sorbitol, and fermentative metabolisms are up-regulated in the endosperm center. This spatial metabolic regulation allows the production of ATP needed for the significant zein synthesis that occurs at the endosperm periphery; this finding agrees with the zein-decreasing gradient previously observed from the sub-aleurone layer to the endosperm center. The massive synthesis of proteins transiting through endoplasmic reticulum elicits the unfolded protein responses, as indicated by the splicing of bZip60 transcription factor. This splicing is relatively higher at the center of the endosperm than at its periphery. The biological responses associated with this developmental stress, which control the starch/protein balance, leading ultimately to the formation of the vitreous and floury regions of mature endosperm, are discussed.

## Introduction

Endosperm texture is an important quality trait of maize (*Zea mays* L.), determined by the proportions of vitreous and floury endosperm. Thus, the identification of the physicochemical mechanisms leading to vitreousness is fundamental in terms of both nutritional and agronomical issues. This was mainly achieved by studying different opaque/floury mutants, especially *opaque2* (*o2*), and Quality Protein Maize (QPM), where vitreous endosperm is restored by introgressing “*o2 modifier*” genes (*mo2*) within an *o2* background (Gibbon and Larkins, 2005). These studies highlighted the close relationships between zeins, starch, and maize vitreousness. Zeins, the storage proteins, are synthesized in the endoplasmic reticulum (ER) and stored in protein bodies. Numerous floury/opaque mutants are affected in zein biosynthesis, that leads to defect in the structure and shape of protein bodies (Wu and Messing, 2010). Indeed, *o2* encodes a defective transcription factor (TF) that, in its wild-type form, regulates the expression of several genes during endosperm development. Such mutations result in a decrease in zein content and smaller protein bodies (Schmidt et al., 1987, 1990). *Floury2, floury4, Defective endosperm-B30*, and *Mucronate* mutants display mutations in α-zeins or γ-zein, leading to unshaped protein bodies and low zein content (Coleman et al., 1997; Kim et al., 2004, 2006; Wang et al., 2014). *Opaque1* and *floury1* mutants are impacted in the ER machinery, leading to disrupted protein body shape without a significant decrease in zein content (Holding et al., 2007; Wang G. et al., 2012). Starch biosynthesis is also involved in endosperm vitreousness. Indeed, *opaque5 (o5)*, and *shrunken4* (*sh4*) mutants that are affected in starch biosynthesis also display a floury endosperm (Yu et al., 2001; Myers et al., 2011). Finally, the recovery of a vitreous endosperm in QPM is related to an increase in amylose content and shorter amylopectin branching (Gibbon et al., 2003; Salazar-Salas et al., 2014) as well as an increase in 27 kDa γ-zein content and well-shaped protein bodies (Wu et al., 2010).

However, opaque mutations and *mo2* genes have pleiotropic effects in regard to both the number and differential expression level of up- and down-regulated genes when compared with the wild type (Hunter et al., 2002). Recently, characterization of flint and dent maize inbred lines, used in breeding programs, revealed gradients of proteins and starch closely related to the vitreous/floury transition (Gayral et al., 2016). In mature endosperm, the decrease in protein content from the periphery to the center of the endosperm is mainly attributed to α-zeins. Amylose content follows the same distribution, leading to a starch crystallinity gradient. This starch crystallinity is also affected by higher amounts of starch-bound lipids in the vitreous area (Gayral et al., 2015). These gradients are in agreement with the centrifugal development of endosperm from mitotic cell division to programmed cell death (PCD). In maize, PCD starts at the center of the endosperm (the developing floury region) to finally affect the entire endosperm, including the periphery (the developing vitreous region; Young and Gallie, 2000a,b). The origin of endosperm cell death has not yet been established; nevertheless, it is known that developing maize endosperm endures different stresses. Specifically, endosperm faces hypoxic conditions from 10 days after pollination (DAP; Rolletschek et al., 2005). In fact, oxygen deficiency leads to different responses, which result in plant survival or cell death (Bailey-Serres et al., 2012). Characterization of the molecular processes associated with endosperm development is essential to assist in breeding of conventional maize hybrids for improved vitreousness adapted to food uses. In particular, in the context of climate changes, close relationships may be expected between certain genes expressed during endosperm development and those expressed during abiotic (heat and drought) stress. Therefore, a transcriptomic approach combined with the analyses of key metabolites was performed on maize inbred lines. The results showed that the development of vitreous and floury endosperm is clearly associated with significant differences in the responses to hypoxia and ER stress through the regulation of storage protein biosynthesis and energy metabolism.

## Experimental procedures

### RNA extraction

For RNA extraction we used 10 grains from the same ear of a flint and a dent inbred line grown in the Limagrain research station (Mons, France) and harvested at 15 and 20 DAP. The inbred lines L3 (flint) and L5 (dent) were chosen from a previous study where 13 flint and dent inbred lines were analyzed for their protein, starch, and lipid contents in relation to endosperm vitreousness (Gayral et al., 2015, 2016). Two biological repetitions were carried out with grains from different ears. The grains were hand-dissected. In a first time, the middle third of the grain was isolated with a scalpel blade, then an endosperm piece of tissue was extracted from the central endosperm (which will develop in the floury region) and the peripheral endosperm (which will develop in the vitreous region) using a small cookie cutter (Ø = 0.9 mm), and immediately poured into liquid nitrogen before grinding. Total RNA extraction for microarray and RT-PCR (reverse-transcription PCR) analysis was performed using the RNeasy Plant Mini Kit (Qiagen, Les Ulis, France).

### Statistical analysis of microarray data

Experiments were designed with the Genomics Networks team of the Institute of Plant Sciences (IPS2). For each array, the raw data comprised the logarithm of median intensities of feature pixels at wavelengths of 635 nm (red) and 532 nm (green). For each array, a global intensity-dependent normalization using the loess procedure (Yang et al., 2002) was performed to correct the dye bias. The differential analysis was based on averaging the log-ratios over the duplicate probes and over the technical replicates. Hence, the number of available data for each gene equals the number of biological replicates and is used to calculate the moderated *t*-test (Smyth, 2004). Under the null hypothesis, no evidence that the specific variances vary between probes was highlighted by Limma analysis, and consequently the moderated t-statistic was assumed to follow a standard normal distribution. To control the false discovery rate (FDR), adjusted *p*-values found using the optimized FDR approach described by Storey and Tibshirani (2003) were calculated. We considered the probes with an adjusted *p*-value ≤ 0.05 to be differentially expressed. Analysis was performed using R software. The function SqueezeVar of the library Limma was used to smooth the specific variances by computing empirical Bayesian posterior means. The library kerfdr (kernel-based local fdr) was used to calculate the adjusted *p*-values.

### Data deposition

Microarray data from this article were deposited in the international repository GEO, Gene Expression Omnibus (Edgar et al., 2002; http://www.ncbi.nlm.nih.gov/geo/), accession no. GSE88993. All steps of the experiment, from growth conditions to bioinformatic and statistical analyses, were detailed in CATdb (Complete *Arabidopsis* Transcriptome database; Gagnot et al., 2008; http://tools.ips2.u-psud.fr/CATdb/; Project: 4 plex_Maize_2015_02) according to the “Minimum Information About a Microarray Experiment” standards.

### Annotation and gene ontology

Genes were annotated by sequence homology with *A. thaliana* or *O. sativa* according to MaizeGDB (Maize Genetics and Genomics Database; http://www.maizegdb.org; Sen et al., 2009).

Identification of GO (gene ontology) categories significantly enriched (*p* ≤ 0.05) was based on “plant GO slim” and done using online AgriGO software (http://bioinfo.cau.edu.cn/agriGO; Du et al., 2010).

### qRT-PCR

Total RNA (4 μg) was reverse-transcribed using SuperScript III and PolyT primer (Invitrogen, ThermoFisher Scientific, Courtaboeuf, France). qPCR (qualitative PCR) quantification was performed with CFX Connect (Bio-Rad, Marnes-la-Coquette, France) and Power SYBR Green PCR Master Mix (Applied Biosystems, ThermoFisher Scientific, Courtaboeuf, France). The relative gene expression level was normalized to ubiquitin (GRMZM2G109977), and was defined as ΔCq = 2^∧^−(Cq_Gene_ − Cq_Ubi_). The data represent the mean of two technical replicates for two biological repetitions (± *SD*). All primers used in the study are listed in Table S1.

### Metabolite extraction and quantification

For metabolite extraction, we used five grains (30 DAP) from the same ear. Three biological repetitions were carried out from different ears. Grains were hand-dissected, and samples were freeze-dried and ground in liquid nitrogen. Extraction was conducted on 20 mg of sample with 500 μL of CH_3_OH containing 0.2 mM norleucine and 0.4 mM adonitol as an internal standard. After 15 min of stirring, 250 μL of CHCl_3_ and 500 μL of water were added. After stirring and centrifugation, the aqueous phase was collected and used for metabolite quantification. Aliquots (100 μL) were dried in a SpeedVac concentrator (Savant, ThermoFisher Scientific, Courtaboeuf, France) and derivatized for 2 h at 37°C in 50 μL 20 mg.mL^−1^ methoxyamine in pyridine, then for 30 min at 37°C after the addition of 50 μL of BSTFA (N, O-bis (trimethylsilyl) trifluoroacetamide). Metabolites were analyzed by GC-FID/MS (gas chromatography with flame ionization detection and mass spectrometry) using Clarus SQ8C (Perkin Elmer, Villebon sur Yvette, France) equipped with a DB-5MS capillary column (30 m × 0.25 mm × 0.25 μm; Agilent Technologies, Les Ulis, France), and identified according to mass spectra and retention time compared with standard solution. Metabolites were normalized and quantified according to adonitol.

For quantification of free amino acids, aliquots (200 μL) were dried in a SpeedVac concentrator, and derivatized in 20 μL H_2_O/CH_3_CH_2_OH/N(CH_2_CH_3_)_3_/PITC (phenyl isothiocyanate) (1/7/1/1) for 10 min at room temperature. Samples were then dried and solubilized in 100 μL 2 mM-Na_2_HPO_4_ pH 7.4, with 5% of CH_3_CN. Free amino acids were analyzed by reversed phase HPLC (reversed-phase high-performance liquid chromatography) using a Pico-Tag column (3.9 × 300 mm; Waters, Guyancourt, France) on an Alliance HT apparatus equipped with a UV detector (254 nm; Waters, Guyancourt, France). Elution (1 mL.min^−1^) was performed at 40°C using a linear gradient from 100% eluent A (70 mM-CH_3_ÂČCOONa pH 6.45 with 2.5% CH_3_CN) to 100% eluent B (CH_3_CN/CH_3_OH/H_2_O (45/15/40). The quantity of amino acid was normalized to the norleucine internal standard and quantified in regard to the calibration curve obtained by injection of standard amino acids (20,088, Pierce, ThermoFisher Scientific, Courtaboeuf, France) derivatized under the same conditions.

## Results

### Transcriptome specifications of central and peripheral endosperm during grain filling

**Figure 1:**
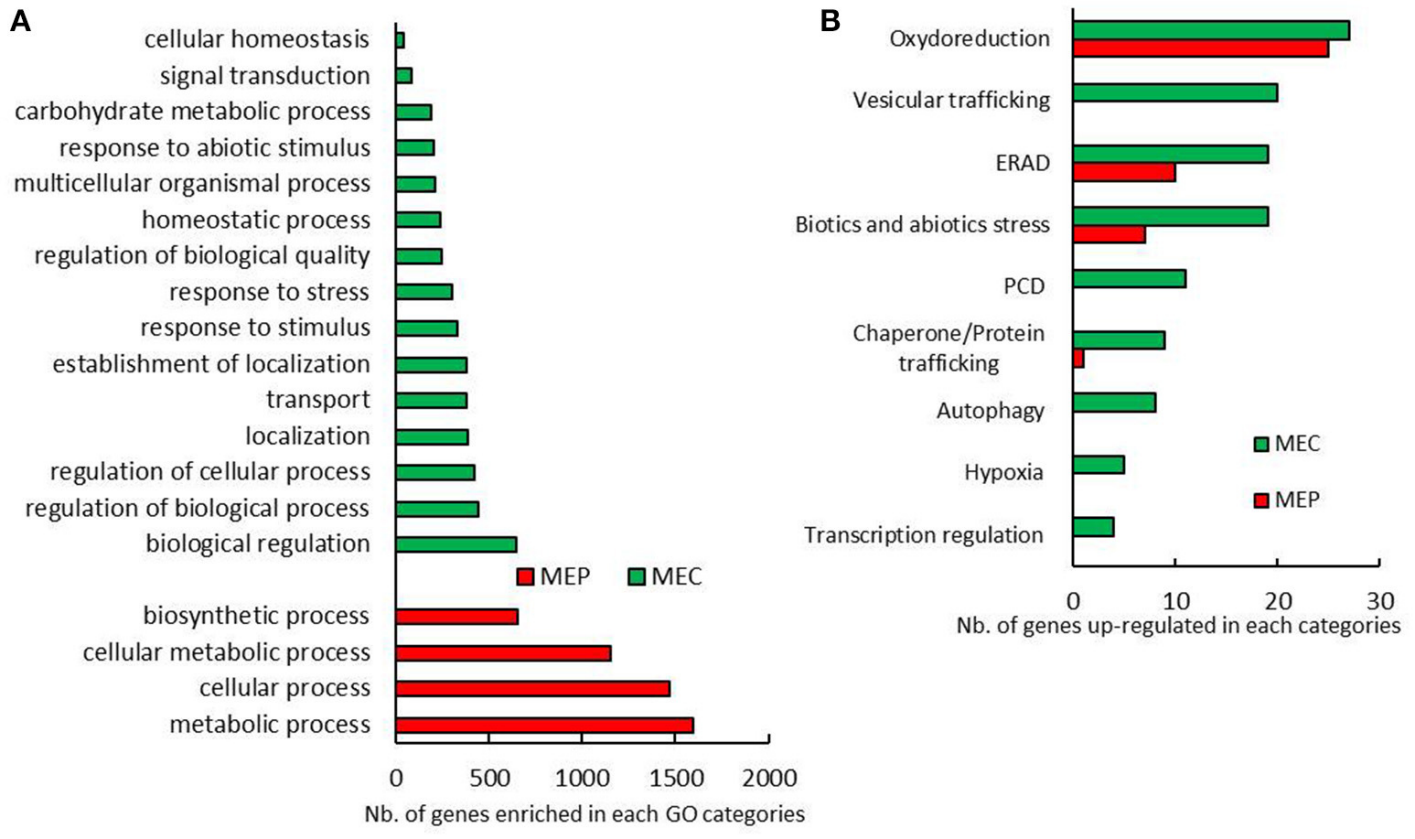
**MEP and MEC transcriptome pattern. (A)** Biological process GO categories significantly enriched in genes only up-regulated in MEC and MEP in almost a tested condition. **(B)** Annotated genes related to stress differentially expressed between MEC and MEP at 15 DAP and 20 DAP in flint and dent maize.

Maize grain development starts with the double fertilization of the female gamete and the central cells, which leads to the development of the embryo and the endosperm respectively. Upon 10 DAP, protein and starch begin to accumulate in the endosperm. To ascertain whether transcriptome modifications are related to vitreousness, we analyzed the RNAs extracted from central and peripheral endosperm that will become at maturity the floury and the vitreous areas, respectively. From here on, we will refer to these regions as maize endosperm center (MEC) and maize endosperm periphery (MEP). MEC and MEP transcriptomes were analyzed on a flint and a dent inbred line harvested at 15 and 20 DAP. By considering only genes up-regulated in MEC, i.e., 5,518, or in MEP, i.e., 5,167, at least in a developing stage and in a genetic background (flint or dent), gene ontology (GO) analysis revealed enrichment in “metabolic process,” “cellular process,” “cellular metabolic process,” and “biosynthetic process” in MEP, whereas in MEC we notably observed enrichments in “response to stress,” “response to stimulus,” and “response to abiotic stimulus” (Figure 1A). These results suggest that MEC and MEP follow their own development program, especially with respect to the stronger stress responses in MEC.

From 15 to 20 DAP developing stages, at least 9369 genes were found to be differentially expressed when MEC and/or MEP of flint and/or dent inbred lines were compared. Considering genes significantly regulated in MEC and MEP, we did not observe strong GO enrichment pattern differences (Figure S1A). Enriched GO categories are mainly related to metabolic and biosynthetic processing (Figure S1A), in agreement with the massive synthesis of storage compounds. However, we only observed GO enrichment of “response to stress” in MEC, whereas “generation of precursor metabolites and energy” and “multicellular organismal process” were enriched in MEP. Therefore, MEC and MEP follow a close developmental process, and differences in stress responses observed between MEC and MEP persist throughout grain filling.

To further characterize the relationships between developmental stress responses and maize vitreousness, we focused on genes that showed significant differences in expression levels between MEC and MEP in flint and dent endosperm at 15 and 20 DAP: 634 genes were up-regulated in MEP at 15 and 20 DAP in flint and dent inbred lines, and 525 genes were annotated, while in MEC, 776 genes were up-regulated and 602 were annotated. Differentially expressed genes involved in stress management are shown in Figure 1B. In MEP, the expression of genes involved in oxido-reduction (including responses to oxidative burst), ER-associated degradation (ERAD) and abiotic/biotic stress responses was clearly activated. In MEC, we also observed the expression of genes involved in vesicular trafficking, PCD, autophagy, hypoxia, and regulation of transcription (Figure 1B); these data suggested a higher stress response in this area at 15 and 20 DAP.

To further investigate the temporal modifications linked to vitreousness, we focused on genes showing significant differences in MEP and/or MEC in both flint and dent lines between 15 and 20 DAP. 378 genes were significantly up-regulated from 15 to 20 DAP in MEP and/or MEC in the two inbred lines, and 258 of these genes were annotated. On the other hand, 348 genes were found to be significantly down-regulated (from 15 to 20 DAP) in MEP and/or MEC in the two inbred lines, and of these genes, 280 were annotated. If we consider the genes involved in stress responses, their expression was mainly up-regulated at 20 DAP (Figure S1B). In fact, almost the same genes were found to be up-regulated in MEP and MEC, with the exception of those implicated in ERAD, essentially up-regulated in MEC. Between 15 and 20 DAP, PCD genes were found to be up-regulated in both MEC and MEP (Figure S1B), confirming that PCD increases throughout endosperm development as previously shown (Young and Gallie, 2000a,b).

### Starch and zein-associated transcriptome signatures

In regard to the genes encoding enzymes involved in core starch biosynthesis, ADP-glucose pyrophosphorylase, starch synthases, branching, and debranching enzymes, we did not observe any coherent differential expression. Indeed, certain genes were found to be up-regulated in MEP, while their homologs were found to be up-regulated in MEC (Figure S2). These findings are probably due to the fact that starch deposition follows gradients from the upper to the basal part of the endosperm (Dochlert, 1990) as well as from the periphery to the center (Gayral et al., 2016). This fact could also be related to the regulation of starch biosynthesis at the post-translational level through phosphorylations and formation of multienzyme complexes (Hennen-Bierwagen et al., 2009). In fact, starch biosynthesis starts with ADP-glucose a product provided by the sucrose synthase (SUS) pathway. Indeed, ectopic overexpression of a potato SUS in maize induces an increase in ADP-glucose and starch contents (Li et al., 2013). Three *sus* genes are strongly expressed in maize endosperm: *sus1* (GRMZM2G152908), *sus2* (GRMZM2G318780), and *sus-sh1* (GRMZM2G089713; Figure S2). Previous studies suggest that SUS1 and SUS-SH1 are involved in starch and cell wall polysaccharide synthesis, respectively (Chourey et al., 1998). In contrast with invertases, SUS is an energy-conserving pathway for sucrose breakdown since it uses one PPi molecule and produces one UTP molecule. UTP can be used to phosphorylate fructose- and subsequent entry of fructose-6-phosphate in the glycolysis pathway (Geigenberger, 2014). *Sus1* (GRMZM2G152908) and *sus2* (GRMZM2G318780) are up-regulated in MEP (Figure S2), and display the highest differential expression among all genes that discriminate developing MEP from developing MEC. This is consistent (i) with the localization of the intense corresponding activity at the periphery of developing maize endosperm (Wittich and Vreugdenhil, 1998) (ii) the formation of SUS1-SUS2 hetero-oligomers (Duncan et al., 2006) and (iii) the up-regulation of *sus1* in hypoxic conditions (Zeng et al., 1998). Any differential expression is observed for *sus-sh1* (GRMZM2G089713) that encodes SUS-SH1 (Figure S2) in agreement with the fact that the expression of this gene is not up-regulated by hypoxia but by anoxia, a huge depletion of oxygen (Zeng et al., 1998). However, up-regulation of *sus1* and *sus2* in MEP is contradictory to (i) slightly lower starch contents in the vitreous than in the floury endosperm (Gayral et al., 2015) and (ii), as shown below, to up-regulation of genes markers of hypoxia in MEC. Altogether these results suggest that the management of hypoxia differs between MEP and MEC.

**Figure 2:**
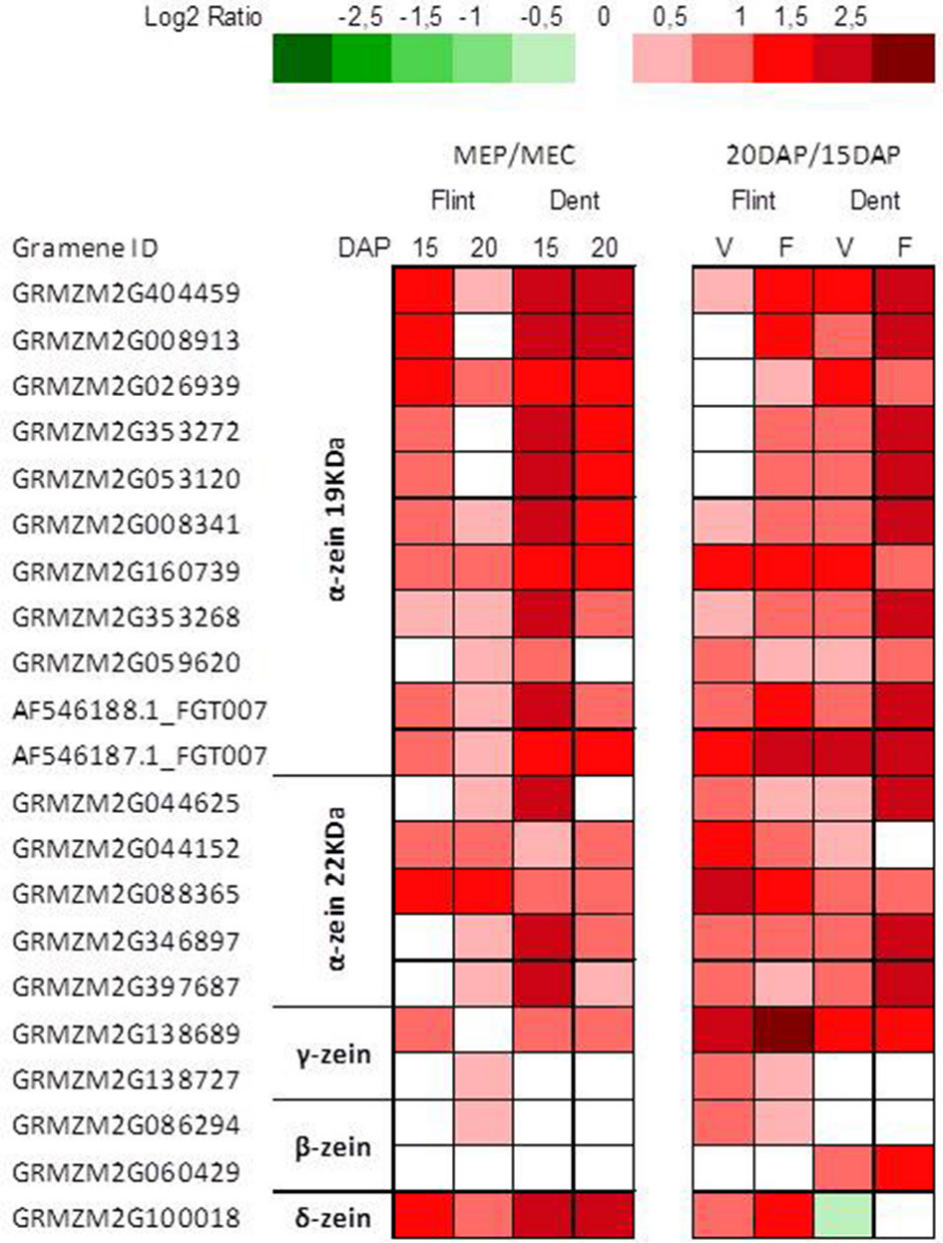
**Transcriptome patterns of zein genes**. Log_2_ ratio between MEP (V) and MEC (F) for genes specifying zeins at 15 and 20 DAP in flint and dent inbred lines. Genes ID and log_2_ ratio are listed in Table S3.

In contrast with starch deposition, protein deposition is significantly higher in the periphery of the endosperm (Gayral et al., 2016) than in its longitudinal axis, i.e., from the upper to the lower part of the endosperm (Dochlert, 1990). Furthermore, expression of zein genes is essentially regulated at the transcriptional level (Zhang et al., 2015; Qiao et al., 2016). Indeed, transcriptomic analysis revealed stronger zein expression in MEP at 15 and 20 DAP, especially for α and δ-zeins (Figure 2). These results agree with those of mRNA *in-situ* hybridization experiments, which revealed stronger α-zein expression at the periphery of developing endosperm (Woo et al., 2001). Furthermore, we observed a global up-regulation of genes specifying zeins at 20 DAP (Figure 2), in accordance with epigenetic regulation of α-zein expression in endosperm at 15–23 DAP (Locatelli et al., 2009). These results are consistent with the protein decrease observed previously from the sub-aleurone layer to the endosperm center (Gayral et al., 2016).

### ER-stress, UPR and endosperm development

**Figure 3:**
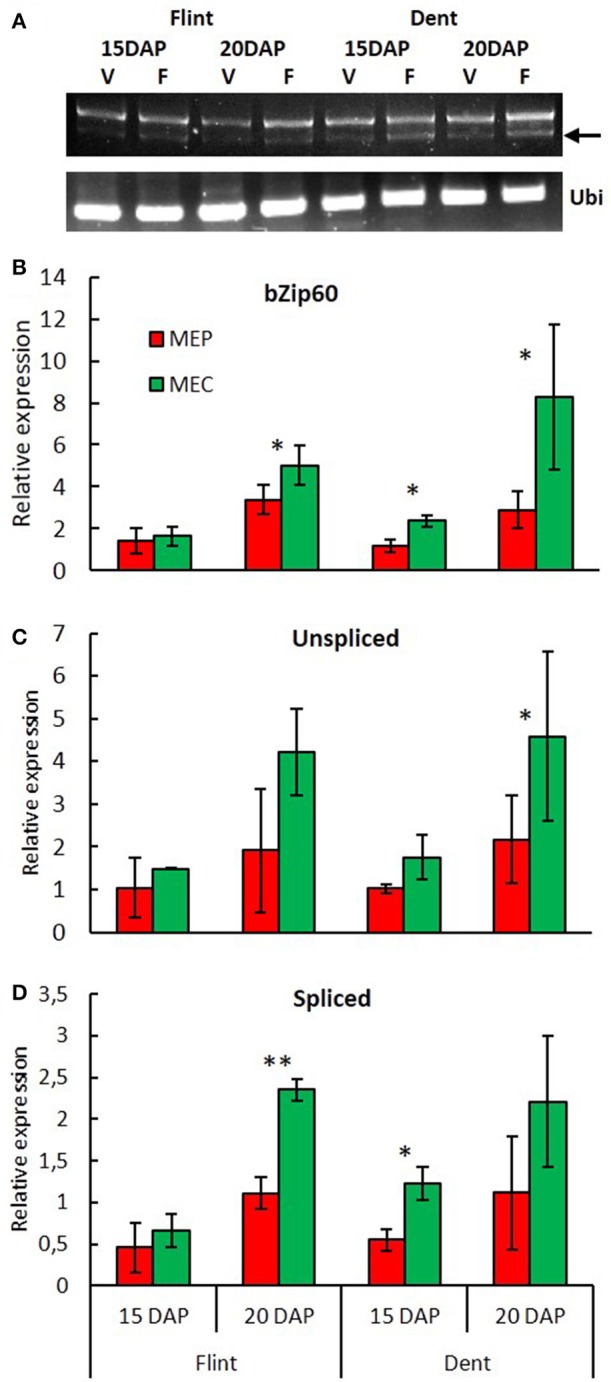
**Spatiotemporal evolution of UPR during endosperm development. (A)** *bzip60* (GRMZM2G025812) splicing was detected by RT-PCR in MEP and MEC at 15 and 20 DAP. Ubi: RT-PCR of *ubiquitin* (GRMZM2G109977). Arrow: spliced mRNA. Relative expression of *bzip60* **(B)**, unspliced *bzip60* **(C)** and spliced *bzip60* **(D)** measured by qRT-PCR in MEP and MEC at 15 and 20 DAP. Relative expression level was normalized to *ubiquitin* (GRMZM2G109977). Asterisks indicate significant difference (paired *t*-test; ^*^p < 0.05; ^**^p < 0.01).

Down-regulation of genes specifying zeins in MEC is associated with up-regulation of genes involved in stress responses (Figure 1). When checked, chaperones, protein degradation (ERAD), vesicular trafficking, autophagy, and the transcription process are up-regulated in MEC (Figure 1B). All these mechanisms are generally induced in response to ER stress, leading to the corresponding signaling cascade named the unfolded protein response (UPR) and suggest stronger UPR in MEC than in MEP. In plants, two UPR pathways have been described. The first UPR pathway involves bZIP17/bZIP28, i.e., two membrane-associated TFs that are exported from ER to Golgi apparatus en route to the nucleus; the second pathway involves IRE1, i.e. a dual-protein kinase, and the bZIP60 TF. When unfolded or misfolded proteins accumulate in the ER, IRE1 is activated and the *bzip60* mRNA is spliced (Howell, 2013). The spliced form of *bzip60* leads to mRNA encoding a TF with a canonical nuclear targeting signal (Li et al., 2012; Wang B. et al., 2012). In addition to promoting UPR genes, bZIP60 up-regulates its own expression. In fact, microarray analysis showed a higher expression of *bzip60* in MEC than in MEP for both flint and dent maize and at both 15 and 20 DAP (Table S2). To characterize the UPR level in MEC and MEP, we followed *bzip60* splicing during endosperm development by quantifying the unspliced and spliced forms of this TF. RT-PCR analyses revealed spliced mRNA in both MEC and MEP at 15 and 20 DAP for both flint and dent maize (Figure 3A), attesting to the fact that UPR took place in developing endosperm. In flint, and especially in dent, inbred lines, qRT-PCR analyses globally showed a higher expression level at 20 DAP (Figure 3). These results reflect the increase in stress during endosperm development, as shown in Figure S1. In terms of total *bzip60* and unspliced *bzip60* expression levels, a higher level was observed in MEC, confirmed by microarray analysis (Table S2). When compared, splicing of *bzip60* is more pronounced in MEC than in MEP, confirming a stronger UPR in this region. This difference was found to be more acute at 20 DAP. It is interesting to note that ER stress was previously associated with higher content of triacylglycerols (TAG) in maize endosperm of the *floury2* mutant (Shank et al., 2001), in accordance with higher amounts of these storage lipids in the floury regions of conventional maize (Gayral et al., 2015). The higher TAG deposition in MEC relative to that in MEP is also related to major changes in the proteins that cover and stabilize oil droplets. Indeed, while an oleosin-like protein is highly up-regulated in MEP (GRMZM2G480954), an ortholog of *Arabidopsis* lipid droplet-associated proteins (LDAP) (GRMZM2G150367) is one of the most up-regulated proteins in MEC. These LDAPs are associated with stress responses and are mostly expressed in vegetative tissues (Laibach et al., 2015). Therefore, ER stress and UPR could be responsible for TAG accumulation in the floury regions of maize endosperm. Taken together, these results reveal a permanent ER stress in the endosperm, with a stronger UPR in MEC than in MEP.

### From endosperm stress responses to autophagy and PCD

UPR evolution is associated with spatiotemporal up-regulation of autophagy and PCD (Figure 1B and Figure S1), suggesting a possible relationship between these processes, especially in MEC. Such a relationship controls the pro-life and pro-death evolution of cells and is commonly encountered in all eukaryotes (Suzuki et al., 2017). Indeed, it is worth noting that the gene specifying BAX inhibitor-1, an ER-resident transmembrane protein that attenuates PCD owing to ER-stress in *Arabidopsis* (Watanabe and Lam, 2008), is up-regulated in MEC (Table S2). In the same way, the gene specifying Bcl-2-associated athanogene 1 (BAG1) is up-regulated in MEC (Table S2). In *Arabidopsis*, BAG1 acts as a co-chaperone in various processes related to environmental stress and PCD (Nawkar et al., 2016). The DAD1 (defender against cell death) gene, also localized in the ER, is another regulator of cell death. *dad1* is well expressed in both MEC and MEP, with a slight tendency to be more up-regulated in MEC (Table S2). DAD1 is a glycosyltransferase that is known to mediate, in *Arabidopsis*, N-glycosylation of nascent proteins and to suppress cell death induced by abiotic stresses (Danon et al., 2004). From 15 DAP, expression of the genes specifying these proteins, in maize endosperm, as well as their up-regulation in MEC, confirm the early PCD observed in this area. Nine metacaspases have been identified in *Arabidopsis*, AtMC1 to AtMC9, and their homologs, i.e., ZmMC1 to ZmMC9, were found in maize (Minina et al., 2013). Though these proteins differ from their animal counterparts, plant metacaspases are markers of PCD (Lam and del Pozo, 2000; Tsiatsiani et al., 2011; Fagundes et al., 2015). Microarray analyses revealed that two type II metacaspases are differentially expressed in maize endosperms: *mc7* is up-regulated in MEC, while *mc9* is up-regulated in MEP (Table S2), suggesting different PCD pathways in these two areas. Indeed, in Norway spruce, a close relationship was established between a type II metacaspase, autophagy and the subsequent PCD pathway (Minina et al., 2013).

Autophagy is a ubiquitous complex process allowing the degradation of cell organelles and the recycling of these components. Many genes encoding autophagy-related proteins (ATG proteins) have been identified (Li et al., 2015), most of them being expressed during endosperm development, suggesting that the autophagic recycling machinery is fully active (Table S2). Remarkably, although differential expression of these genes between MEC and MEP is not systemically evidenced, some genes are up-regulated in MEP as *atg4b*, while *atg6a* and *atg8c* are up-regulated in MEC. In fact, most *atg8* genes, i.e., *atg8b, atg8c, atg8d*, and *atg8e* tend to be up-regulated in MEC (Table S2). ATG8 proteins are essential in the autophagic process since their lipidation by phosphatidylethanolamine is the actual *primum movens* of the autophagic process. ATG12, which participates in ATG8 lipidation, is also essential for autophagy (Suzuki et al., 2017), and consequently *atg12a* is up-regulated in MEC (Table S2). Expression of all *atg* genes confirms that an autophagic process is implicated in maize endosperm development (Chung et al., 2009), and suggests that a stronger autophagy takes place in MEC. These results agree with spatiotemporal relationships between UPR, autophagy and PCD in this endosperm area and show that UPR corresponds with the spatiotemporal development of PCD in maize endosperm.

### Hypoxia response in maize endosperm

#### Expression of hypoxia-responsive genes

Transcriptomic analyses revealed an up-regulation of genes implicated in hypoxic responses in MEC (Figure 1B). Numerous TF homologs to *Arabidopsis* hypoxia-induced TF are up-regulated in MEC. Notably, VII ethylene response factors (VII ERFs), that plays a major role in the response to low oxygen in *Arabidopsis* (Bailey-Serres et al., 2012). VII ERF homologs are found in maize, especially the homolog of RAP2.12/RAP2.2, which is up-regulated in MEC (Table S2). RAP2.12/RAP2.2 induce the expression of different hypoxia-responsive genes as *alcohol dehydrogenase* (*adh*) and *pyruvate decarboxylase* (*pdc*; Bailey-Serres et al., 2012). These hypoxia-induced enzymes were indeed found to be up-regulated in MEC (Table S2).

#### Soluble sugars and free amino acids

**Figure 4:**
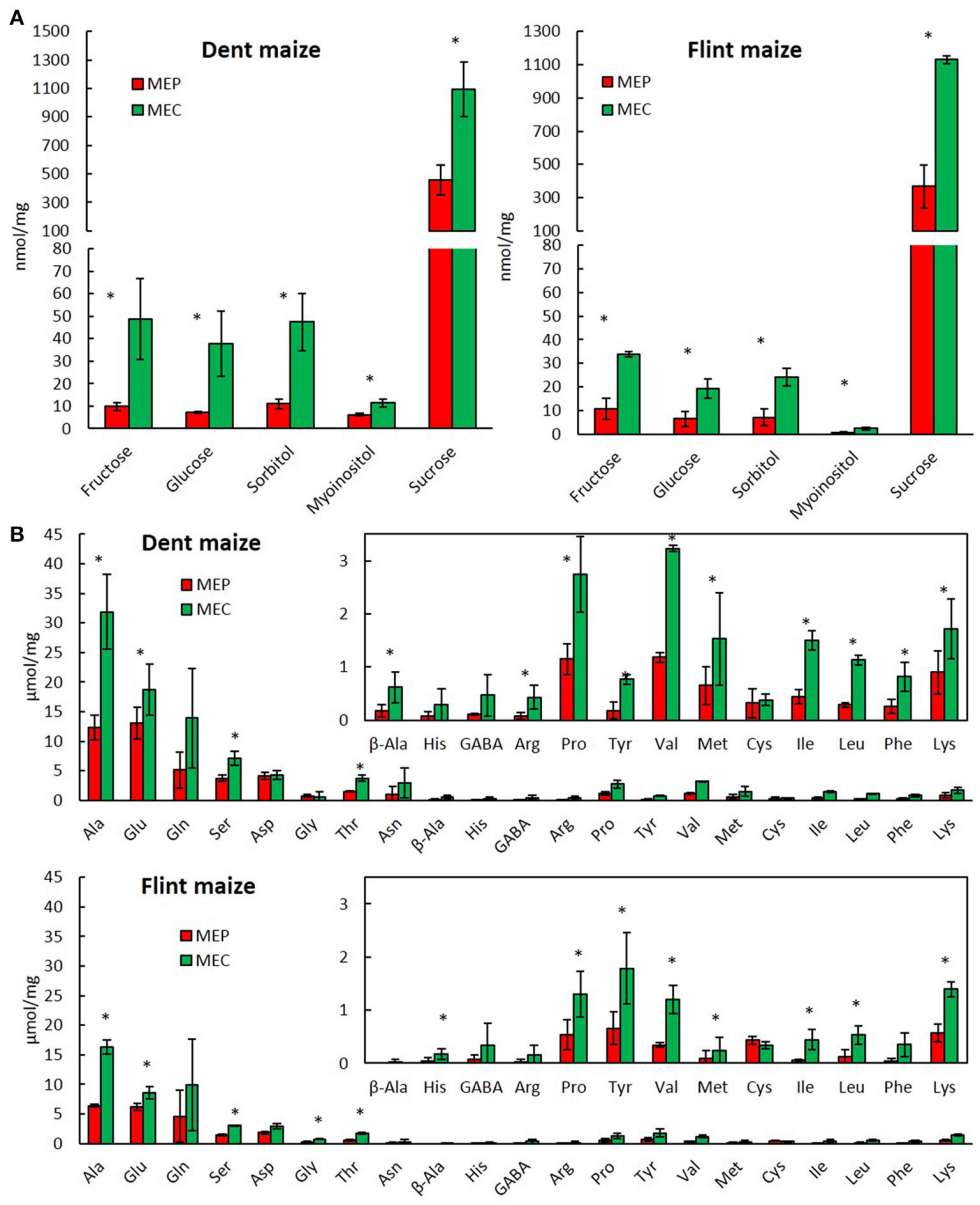
**Metabolite profiling of MEP and MEC**. Soluble sugars **(A)** and free amino acids **(B)** of MEP and MEC in dent and flint maize at 30 DAP. Asterisks indicate significant differences (paired *t*-test; *p* < 0.05).

Analysis of soluble sugars revealed high levels of sucrose in flint and dent endosperm (Figure 4A) consistent with their import from maternal tissue in order to produce starch. In fact, these sucrose levels decrease from the basal to the upper endosperm (Doehlert and Kuo, 1990), following exactly the opposite gradient observed for starch accumulation (Dochlert, 1990). Therefore, the higher amount of soluble sugars in MEC is obviously in total discordance with the higher amounts of starch observed in the floury endosperm (Gayral et al., 2015). Conversely, the lower amounts of almost all free amino acids found in MEP, when compared with those in MEC (Figure 4B), are quite consistent with the significant level of protein synthesis in MEP, leading to a higher protein accumulation in the vitreous region of mature endosperm (Gayral et al., 2016). In flint and dent maize, our results showed high accumulation of alanine, glutamine, glutamate, and serine in whole endosperm, in full accordance with the hypoxic conditions already described (António et al., 2016). In fact, the production of alanine contributes to the preservation of carbon derived from glycolysis in competition with ethanol fermentation under hypoxic conditions. Consequently, the higher alanine amount found in MEC (Figure 4B) supports the hypothesis of a stronger hypoxia (António et al., 2016) affecting this endosperm region.

#### Differential regulation of respiratory and fermentative metabolisms in MEC and MEP

**Figure 5:**
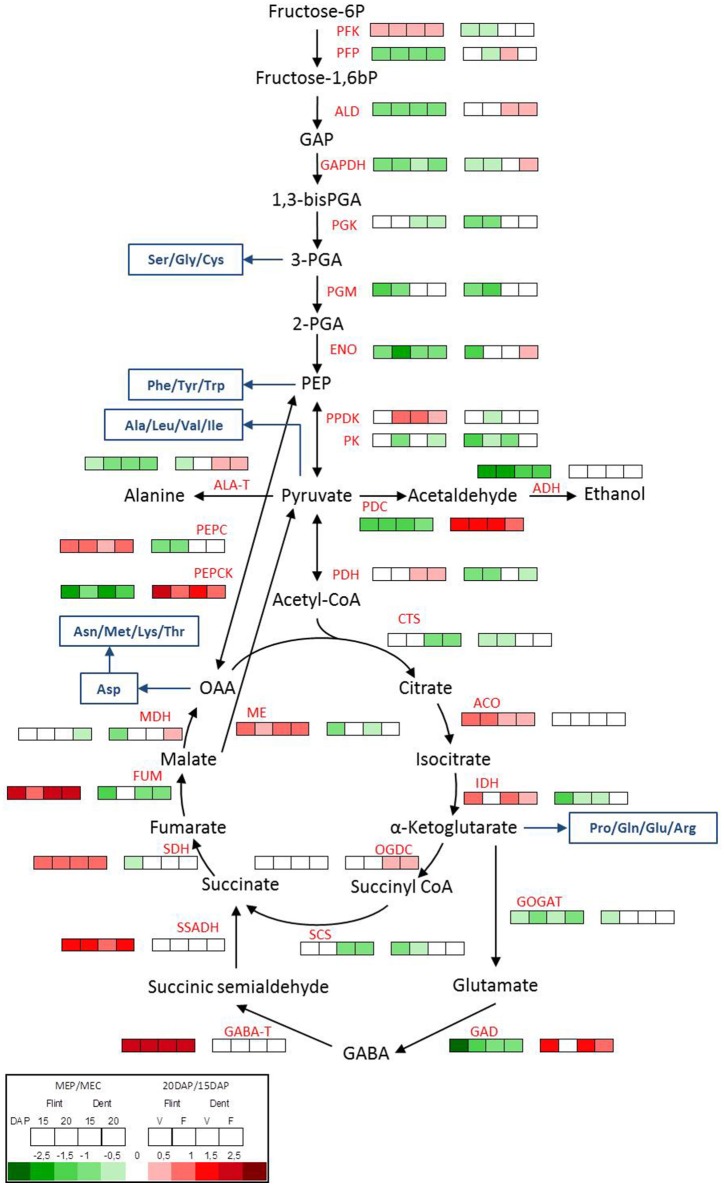
**Metabolic switch between MEP and MEC**. Transcriptome pattern of genes involved in glycolysis, fermentation and TCA cycle in MEP (V) and MEC (F) at 15 and 20 DAP in flint and dent inbred lines. Right box lines represent MEP/MEC log_2_ expression ratio. Left box lines represent 20/15 DAP log_2_ expression ratio. For multigenic families, the results represent the mean of all genes. Genes ID and log_2_ ratio of all genes are listed in Table S3. Blue lines, amino acid synthesis pathway; PFK, phosphofructokinase; PFP, pyrophosphate-fructose-6-phosphate-1-phosphotransferase; ALD, fructose-bisphosphate aldolase; GAPDH, glyceraldehyde-3-phosphate dehydrogenase; PGK, phosphoglycerate kinase; PGM, phosphoglycerate mutase; ENO, enolase; PPDK, pyruvate orthophosphate dikinase; PK, pyruvate kinase; PDH, pyruvate; PEPC, phosphoenolpyruvate carboxylase; PEPCK, phosphoenolpyruvate carboxykinase; PDC, pyruvate decarboxylase; ADH, alcohol dehydrogenase; ALA-T, alanine aminotransferase; CTS, citrate synthase; ACO, aconitase; IDH, isocitrate dehydrogenase; OGDC, oxoglutarate dehydrogenase complex; SCS, succinyl-CoA synthetase; SDH, succinate dehydrogenase; FUM, fumarate hydratase; MDH, malate dehydrogenase; ME, malic enzyme; GOGAT, glutamate synthase; GAD, glutamate decarboxylase; GABA-T, γ-aminobutyric acid trans-aminase; SSADH, succinate-semialdehyde dehydrogenase.

Hypoxia is directly linked to changes in energy metabolism, particularly to a switch from an oxidative to a fermentative metabolism (Zabalza et al., 2009). To investigate whether this switch is involved in the formation of vitreous endosperm, we analyzed the expression of genes implicated in the glycolytic and fermentative pathways in MEP and MEC (Figure 5 and Table S3). In almost all tested conditions (in flint and dent endosperm at 15 and 20 DAP), the results revealed an up-regulation of glycolytic enzymes from fructose-6-P to pyruvate in MEC (Figure 5). Moreover, *pdc* and *adh* are clearly up-regulated in this area at 15 and 20 DAP. Ethanol fermentation thus ensures the NAD (nicotinamide adenine dinucleotide) supply required for glycolysis, allowing energy production under hypoxic conditions. The sorbitol dehydrogenase (SDH) pathway can also generate NAD during conversion of fructose to sorbitol. The gene specifying SDH is slightly up-regulated in MEC, which correlates with accumulation of sorbitol in this region of the endosperm (Figure 4A and Figure S2) and its up-regulation by oxygen depletion (de Sousa et al., 2008). Furthermore, in MEC, we also observed an up-regulation of *alanine aminotransferase* (*ala-t*), which fully concurs with the higher alanine content of the floury endosperm (Figure 4).

It has been shown that pyrophosphate-fructose-6-phosphate1-phosphotransferase (PFP) and pyruvate orthophosphate dikinase (PPDK) activities are related to vitreousness recovery in QPM (Guo et al., 2012), the latter may potentially play a role in starch/protein balance (Mechin et al., 2007; Prioul et al., 2008a). During glycolysis, fructose-6-P is phosphorylated by phosphofructokinase (PFK) or PFP. Interestingly, *pfp* is up-regulated in MEC, whereas *pfk* is slightly up-regulated in MEP (Figure 5). Actually, PFK uses ATP whereas PFP uses PPi, allowing thus to save ATP under hypoxic conditions. These results fully concur with the stronger hypoxic conditions in MEC. Downstream of glycolysis, the expression of genes specifying pyruvate kinase (PK) and PPDK both producers of pyruvate from phosphoenolpyruvate (PEP), did not have the same transcriptomic pattern (Figure 5). Whereas *pk* was rather up-regulated in MEC, *ppdk* was slightly up-regulated in MEP, which correlates with QPM vitreousness adaptation. In the next step, pyruvate is catalyzed by pyruvate dehydrogenase (PDH). Conversely to the previous step of glycolysis, *pdh* was found to be weakly up-regulated in the MEP of dent maize and remarkably stable in flint maize. This result confirmed the close relationship already observed between acetyl-CoA and oxygen availability (Rolletschek et al., 2005).

All these results show that both respiratory and fermentative pathways co-exist in MEC and MEP, but in MEC, glycolysis is rather oriented toward fermentation and alanine production, whereas in MEP, glycolysis is rather directed to the TCA cycle to ensure ATP production. In the same way, the gene specifying phosphoenolpyruvate carboxylase (PEPC), which catalyzes the decarboxylation of pyruvate to oxaloacetate (OAA), is up-regulated in MEP, whereas the gene specifying phosphoenolpyruvate carboxykinase (PEPCK), which can catalyze the reverse reaction, is up-regulated in MEC (Figure 5). In view of the fact that citrate synthesis from PEP via PEPC is active in maize kernel (Jeanneau et al., 2002), the expression pattern of *pepc* and *pepck* also suggests a higher fermentative metabolism in MEC. In MEP, a part of the pyruvate is probably phosphorylated, leading to PEP in order to produce PPi, an energy source substitute of ATP under low oxygen conditions. The produced PEP is then probably transferred to TCA cycle through PEPC. In MEC however, OAA could be carboxylated to generate PEP, which could be used for the fermentative metabolism requirements. Interestingly, both *pdc* and *pepck* are strongly up-regulated between 15 and 20 DAP (Figure 5), suggesting a stronger fermentative metabolism at 20 DAP, which fully concurs with the stronger hypoxic conditions. All these results suggest that a fermentative metabolism starts earlier in MEC than in MEP.

### Differential regulation of TCA cycle and GABA shunt in MEC and MEP

The first step in the TCA cycle is the production of citrate catalyzed by citrate synthase (CTS). The gene specifying CTS is up-regulated in MEC in dent maize. Subsequently, aconitase (ACO) and isocitrate dehydrogenase (IDH) produce α-ketoglutarate using citrate. In flint and dent inbred lines, the results revealed the up-regulation of *aco* and *idh* expression in MEP (Figure 5). During the TCA cycle, α-ketoglutarate is decarboxylated by the 2-oxoglutarate dehydrogenase complex (OGDC), leading to succinyl CoA and NADH, the succinyl CoA being subsequently hydrolyzed to succinate by succinyl-CoA synthase (SCS). The *ogdc* gene does not display significant differential expression levels in MEP and MEC, while the *scs* gene is up-regulated in the MEC of dent endosperm (Figure 5). Succinate dehydrogenase complex (SDH, also known as complex II of the mitochondrial electron transport chain) catalyzes the oxidation of succinate to fumarate, which is then hydrated, *via* fumarase (FUM), leading to the production of malate. *sdh* and *fum* genes are both clearly up-regulated in MEP (Figure 5). All these results support the hypothesis that maintaining a functional TCA cycle is probably a key step in the formation of vitreous endosperm. Moreover, the up-regulation of *sdh* increases both the net output of NADH and FADH as substrates for the electron transport chain and the direct input of electrons into this chain *via* SDH itself. These results support the idea of a metabolic switch when MEC and MEP are compared, where the latter maintains a significant respiratory metabolism.

Under hypoxic conditions, α-ketoglutarate is also used by glutamate synthase (GOGAT) and glutamate decarboxylase (GAD), which respectively produce glutamate and GABA via the GABA shunt pathway, leading to accumulation of glutamate and GABA (António et al., 2016). The genes specifying GOGAT and GAD are up-regulated in MEC (Figure 5), which concurs with the higher amount of glutamate in this tissue area (Figure 4). Hypoxia leads to a pH decrease (Felle, 2005) since protons are consumed during GABA production. The up-regulation of *gad* in MEC also supports stronger hypoxia in this area. The GABA shunt leads ultimately to succinate production *via* γ-aminobutyric acid trans-aminase (GABA-T) and succinic semi-aldehyde dehydrogenase (SSADH). The genes specifying these enzymes are clearly up-regulated in MEP (Figure 5). These results suggest that in MEP, the GABA shunt leads to the production of succinate and NADH production necessary to maintain the TCA cycle and respiration, respectively. These differences in the regulation of GABA shunt enzymes testify in favor of differences in hypoxia tolerance between MEP and MEC.

## Discussion

After their differentiation, maize endosperm cells start a cellular program allowing the storage of nitrogen (zeins) and carbon (starch). Deposition of these storage compounds follows gradients from the periphery to the center of the endosperm (Gayral et al., 2016) closely related to cell size and ploidy gradient generated by mitosis and endoreplication (Dante et al., 2014). Remarkably, in conventional maize the transition from a floury to a vitreous endosperm also follows a centrifugal gradient (Gayral et al., 2016). In this work, the metabolic specificities of developing central and peripheral endosperms were characterized and useful data were collected in order to delineate the molecular mechanisms that lead to the formation of floury and vitreous endosperm. This was achieved by using differential transcriptomics of MEP and MEC, completed by analysis of some key metabolites. Finally, by performing this work on a flint and a dent maize inbred line, it was possible to target only specific metabolic features of cells that will form, later in the course of development, the vitreous and floury regions of the endosperm independently from the genetic background.

Transcriptomic analyses revealed significant differences between MEP and MEC that persist throughout endosperm development. Therefore, MEP and MEC display different development kinetics in agreement with the corresponding gradient of cell division and growth during endosperm development. Most of these differences were observed in both flint and dent maize inbred lines despite the fact that their genetic backgrounds are significantly different (Unterseer et al., 2016).

These differences are clearly associated with how the endosperm endures various stresses. Hypoxia is probably the *primum movens* of endosperm stress responses. Indeed, hypoxia, previously characterized during maize endosperm development (Rolletschek et al., 2005), was confirmed during the present work, through the expression of different hypoxia-induced genes. In particular, hypoxia induces an oxidative stress, i.e., the production of reactive oxygen species (ROS). ROS act as signaling molecules and lead to strong modifications of energy metabolism to ensure energy supply (Fukao and Bailey-Serres, 2004). This situation generally leads to a metabolic switch from respiration to fermentation in the endosperm of maize (Prioul et al., 2008b), rice (Xu et al., 2008), and wheat (Zhen et al., 2016) during grain filling process. In the present work, a spatial metabolic adaptation between MEP and MEC is suggested when oxygen deprivation is marked throughout the endosperm (Rolletschek et al., 2005). Our results suggest that this adaptation is different between the periphery and the center of maize endosperm. Indeed, in MEP *sus* and GABA shunt up-regulation allows sufficient glycolysis and TCA cycle activities to sustain the ATP and NADH synthesis while, in MEC, fermentative and sorbitol dehydrogenase pathways are clearly up-regulated, ensuring thus a sufficient supply of NAD for glycolysis to allow energy production under hypoxic conditions. Consequently, differential transcriptomic results suggest that both respiration and fermentation occur in both MEC and MEP, but with different relative intensities. Indeed, we can make the assumption that respiration could prevail in MEP allowing sufficient production of ATP for the massive synthesis of storage proteins while fermentation could prevail in MEC limiting thus ATP production and protein synthesis. In that scenario, the lower ATP concentration observed in the periphery than in the central endosperm (Rolletschek et al., 2005) should be due to a rapid ATP consumption.

In regard to this metabolic adaptation, a special focus can be addressed here concerning the regulation of the expression of the three SUS isoforms, i.e., SUS1, SUS2, and SUS-SH1. These three genes are highly expressed but *sus1* and *sus2* expressions are up-regulated in MEP while *sus-sh1* expression remains stable. This constant expression of *sus-sh1* throughout the starchy endosperm, confirms previous experiments showing that *sh1* is up-regulated by anoxia (Zeng et al., 1998) and that developing endosperm is not anoxic (Rolletschek et al., 2005). In regard to up-regulation of hypoxia-related genes in MEC, the up-regulation of *sus1* and *sus2* in MEP is rather paradoxical since *sus1* is up-regulated by hypoxia (Zeng et al., 1998). However, it was previously shown that the oxygen concentration is already low in MEP and similar to that found in MEC, i.e., 1.4% of atmospheric saturation (Rolletschek et al., 2005). This means that (i) up-regulation of hypoxia-related genes in MEP reflects differences in the management of hypoxia rather than in the level of oxygen depletion and (ii) another signaling mechanism induces *sus* up-regulation. Actually, it was shown that *sus1* can be also up-regulated by sucrose (Koch et al., 1992) and, therefore, in the peripheral region of the maize endosperm, this could be due to the sucrose flux from phloem. Up-regulation of these two isoenzymes from 15 to 20 DAP could account for the lower sucrose accumulation in MEP than in MEC observed in late developing endosperm stage (30 DAP), in agreement with a higher SUS activity in MEP than in MEC (Wittich and Vreugdenhil, 1998). The higher sucrose degradation in MEP could supply the glycolysis-TCA pathway through the production of fructose in order to sustain the ATP production required for protein synthesis, particularly elevated in this area. This is in accordance with the fact that sucrose up-regulates both the expression of SUS (Koch et al., 1996) and storage proteins (Hattori et al., 1990). An increase of SUS-dependent sucrose breakdown leads to production of UDP-glucose, a substrate that could enter cell wall polysaccharide and starch biosynthesis pathways. Previous studies suggested that SUS1 is rather involved in starch biosynthesis pathway while SUS-SH1 is rather involved in cell wall biosynthesis (Chourey et al., 1998). Furthermore, the ectopic overexpression of a potato SUS in maize induces an increase of ADP-glucose and starch contents as well as of amylose/amylopectin mass ratio (Li et al., 2013). These findings are contradictory with the slightly lower starch contents in the vitreous endosperm when compared with the floury one but are in full agreement with the higher amylose content observed in vitreous maize endosperm (Gayral et al., 2015). Previous proteomic and transcriptomic analyses of glycolysis enzymes revealed the central role of pyruvate metabolism in the formation of a vitreous endosperm (Mechin et al., 2007; Prioul et al., 2008a). Indeed, in MEP, a part of the synthesized pyruvate is probably phosphorylated by PPDK to produce PEP and PPi, leading potentially to an indirect inhibition of starch synthesis. This inhibition could account for the slightly lower amount of starch in the peripheral vitreous than in the central floury mature endosperm. However, up to now the roles of SUS2, of the corresponding SUS1-SUS2 hetero-oligomers (Duncan et al., 2006) and the complexes between SUS-SH1 and enzymes of the amyloplast starch synthesis pathway (Hennen-Bierwagen et al., 2009) are unknown and need to be finely explored in relation with starch and protein syntheses. Finally, our results emphasize the significance of the regulation of the sugar metabolism and especially of the energy metabolism linked to hypoxia in defining the protein/starch balance and *in fine* endosperm vitreousness.

The second type of stress response disclosed during this study is related to ER stress. In eukaryote cells, ER stress and the resulting response (UPR) regulate ER protein synthesis and degradation to limit the accumulation of unfolded/misfolded protein in the lumen (Howell, 2013; Unterseer et al., 2016). The expression of *bzip60* gene, as well as its splicing, a clear hallmark of such response, confirms that a higher UPR is activated in MEC. This process may impact transcription and protein translation, contributing hence to lower zein levels in MEC, which correlates with lower zein accumulation in floury endosperm (Gayral et al., 2016). UPR could also impact lipid metabolism, and especially, as observed in *floury2* (Shank et al., 2001) could be responsible for the higher amounts of storage lipids in the floury regions of conventional maize (Gayral et al., 2015). This was established in previous works showing that UPR is activated in wild-type maize endosperm, being stronger in many opaque mutants (Hunter et al., 2002; Morton et al., 2015). Moreover, ER stress is often associated with ROS (Blokhina and Fagerstedt, 2010) thus promoting a strong up-regulation of genes associated with oxido-reduction metabolism in MEC. Under these conditions, a higher level of hypoxia responses and ROS in MEC aggravates significantly the UPR process. In plants, ER stress also promotes autophagy (Liu et al., 2012; Pérez-Martín et al., 2014; Yang et al., 2016), up-regulated during maize endosperm development (Chung et al., 2009). MEC is characterized by stronger hypoxia, oxidative burst, and UPR. Moreover, in MEC, UPR is associated with precocious PCD. Although, there is no evidence linking UPR to PCD in maize endosperm, *o2* mutants which have stronger UPR display nevertheless a premature PCD (Young and Gallie, 2000a).

To summarize, the formation of endosperm vitreousness is linked to the capacity of the endosperm to respond to a strong developmental stress that is closely linked to oxygen depletion, regulating thus deposition of protein and starch within the maize endosperm. Among the responses to this developmental stress that controls starch/protein balance, in depth investigations should be done, in particular on the spatio-temporal regulation of SUS expression, oxidative and fermentative metabolisms and on the ER stress response, i.e., UPR, in relation with the interaction between both low oxygen and sucrose signaling.

## Author contributions

DM, MHM, SB, LL, and CD contributed to the design of the whole project; SB, LL, and CD produced and characterized the plant material; MG and MD performed metabolite extraction and analyses; MG and KE performed PCR and qPCR analyses; SMB and SP designed and conducted microarray measurements; MG, SMB, and SP performed statistical analyses and gene annotation of microarray data; all co-authors contributed to the interpretation of data. MG, DM, KE, and BB wrote the manuscript

## Funding

This work was supported by grants from the Fond unique interministériel (FUI GranoFlakes, F1204004C). The platform POPS is supported by the Laboratory of Excellence Saclay Plant Sciences (LabExSPS) (ANR-10-LABX-0040-SPS).

### Conflict of interest statement

The authors declare that the research was conducted in the absence of any commercial or financial relationships that could be construed as a potential conflict of interest.
