# Supplementary figures and images for: Responses to Hypoxia and Endoplasmic Reticulum Stress Discriminate the Development of Vitreous and Floury Endosperms of Conventional Maize (Zea mays) Inbred Lines
Source: Front Plant Sci. 2017 Apr 13;8:557. doi: 10.3389/fpls.2017.00557 (PMC5390489; doi:10.3389/fpls.2017.00557)

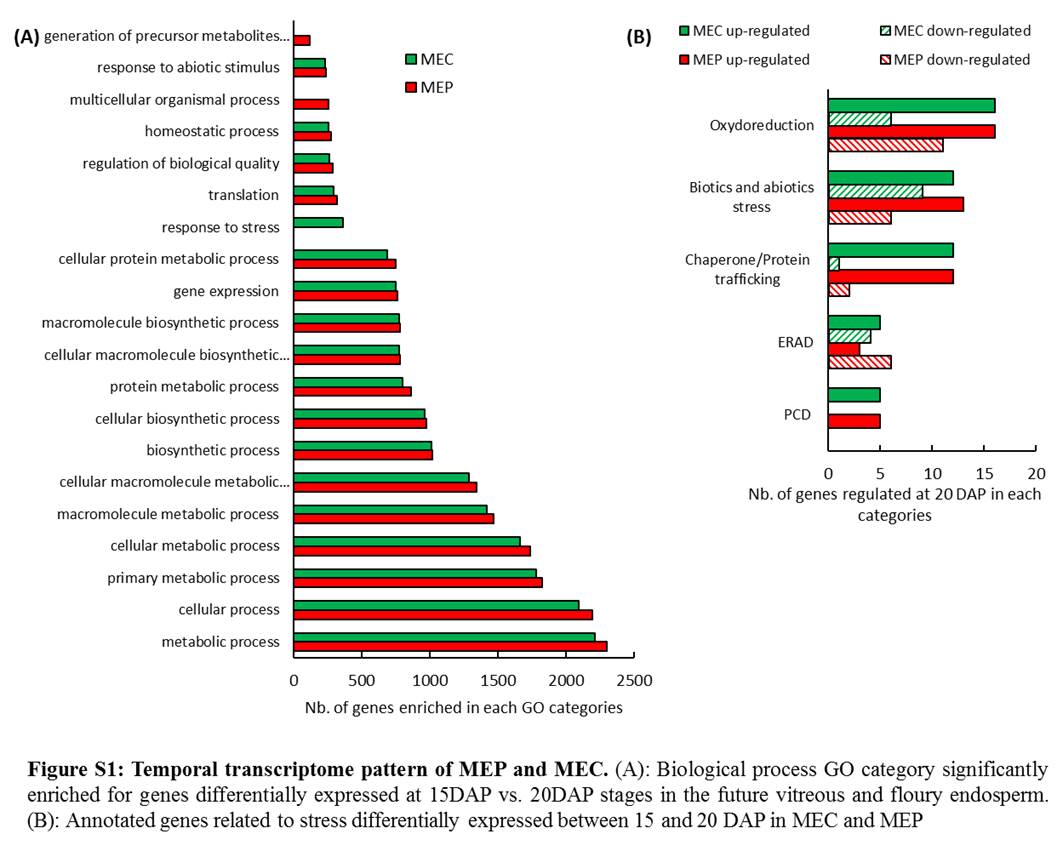

Supplement: Supplementary file 4 [file Image1.JPEG]

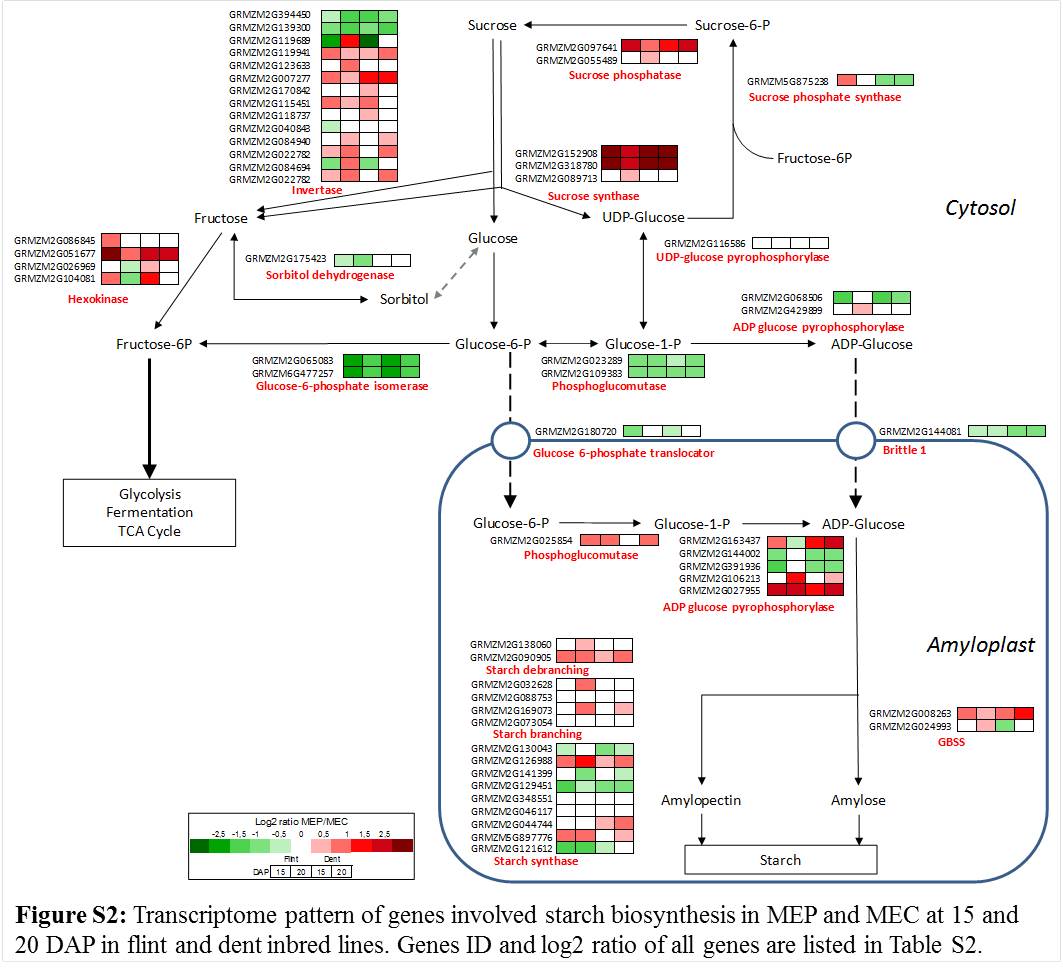

Supplement: Supplementary file 5 [file Image2.JPEG]
